# Putting on the Brakes: Regulatory Kinases and Phosphatases Maintaining B Cell Anergy

**DOI:** 10.3389/fimmu.2018.00665

**Published:** 2018-04-06

**Authors:** S. Elizabeth Franks, John C. Cambier

**Affiliations:** Department of Immunology and Microbiology, University of Colorado Denver School of Medicine, Aurora, CO, United States

**Keywords:** B cells, anergy, SHIP-1, Pten, lyn, SHP-1, phosphatases, kinases

## Abstract

B cell antigen receptor (BCR) signaling is a tightly regulated process governed by both positive and negative mediators/regulators to ensure appropriate responses to exogenous and autologous antigens. Upon naïve B cell recognition of antigen CD79 [the immunoreceptor tyrosine-based activation motif (ITAM)-containing signaling subunit of the BCR] is phosphorylated and recruits Src and Syk family kinases that then phosphorylate proximal intermediaries linked to downstream activating signaling circuitry. This plasma membrane localized signalosome activates PI3K leading to generation of PIP3 critical for membrane localization and activation of plecktrin homology domain-containing effectors. Conversely, in anergic B cells, chronic antigen stimulation drives biased monophosphorylation of CD79 ITAMs leading to recruitment of Lyn, but not Syk, which docks only to bi-phosphorylated ITAMS. In this context, Lyn appears to function primarily as a driver of inhibitory signaling pathways promoting the inhibition of the PI3K pathway by inositol phosphatases, SHIP-1 and PTEN, which hydrolyze PIP3 to PIP2. Lyn may also exert negative regulation of signaling through recruitment of SHP-1, a tyrosine phosphatase that dephosphorylates activating signaling molecules. Alleles of genes that encode or regulate expression of components of this axis, including SHIP-1, SHP-1, Csk/PTPn22, and Lyn, have been shown to confer risk of autoimmunity. This review will discuss functional interplay of components of this pathway and the impact of risk alleles on its function.

## Introduction

The stochastic nature of lymphocyte repertoire diversification leads to production of many B cells that are autoreactive. In fact, it is estimated up to 70% of newly generated B cells recognize self-antigens (1). These autoreactive cells must be silenced to prevent the production of pathogenic autoantibodies and presentation of autoantigen-derived peptides to potentially pathogenic T cells. Silencing of these cells occurs by three known mechanisms. If B cell antigen receptor (BCR) interactions with autoantigen are of high avidity and the cell is immature, antigen receptor signals activate receptor editing, wherein immunoglobulin light chain allele usage changes to an alternative allele (2, 3). If this process eliminates BCR autoreactivity, now harmless cell proceeds to the periphery where it can participate in protective immune responses. If alternate light chain usage does not remove sufficient autoreactivity, continued autoantigen-induced signaling results in apoptotic death, a process known as clonal deletion (4, 5). If remaining autoantigen avidity is significant, but too low to drive receptor editing or clonal deletion, the B cell proceeds to the periphery where it exhibits reduced lifespan and is hyporesponsive to further antigen stimulation, a condition termed anergy (6). Anergic B cells fail to mobilize calcium (7), upregulate activation markers (8), and/or proliferate and differentiate in response to antigen (9).

It seems intuitive that among these silencing mechanisms, anergy is most fragile. Residence in the periphery increases the likelihood that anergic cells encounter inflammatory cytokines, stimulatory pathogen- and damage-associated molecular patterns, PAMPS, and DAMPS that may compromise their unresponsiveness. In addition, maintenance of the anergic state is dependent on continued occupancy of antigen receptors by antigen (10). Removal of autoantigen results in acquisition of responsiveness within minutes (10). This “reversibility” likely confers additional risk of participation in autoimmunity, but is also informative regarding the molecular mechanisms underlying anergy.

The rapid reversibility of anergy suggests that unresponsiveness is maintained by non-durable biochemical pathways and not by genetic reprogramming. Consistent with this possibility, analysis of differences in the transcriptomes of naïve and anergic B cells have failed to reveal mediators of anergic B cell unresponsiveness (11). This review is focused on the molecular regulatory mechanisms that are uniquely induced in anergic cells and are involved in maintenance of their unresponsiveness.

## Characteristics of Anergic B Cells

Naïve B lymphocyte recognition of antigen leads to transduction and propagation of signals that induce expression of activation markers and prepare the cell to interact productively with T cells. However, chronic binding of antigen in the absence of secondary signals provided by T cells, DAMPs, and/or PAMPs, leads to unresponsiveness (12). This anergy is maintained when as few as 20% of receptors are occupied (13), thus unresponsiveness is not caused by inability to bind self or cross-reactive exogenous antigen. Further, this state is not maintained by tonic regulators as it must be induced by antigen receptor stimulation (14–16). Therefore, the mechanisms that maintain the antigen unresponsiveness of anergic B cells can be expected to have the following properties: they require induction by chronic antigen receptor stimulation, they are non-durable, and they affect receptor-proximal signaling events.

Although the concept evolved from studies of normal mice (17), most of what is known about B cell anergy was learned by studying immunoglobulin transgenic mice in which all B cells share reactivity with an autoantigen. Perhaps most notable are MD4 immunoglobulin transgenic mice that express mIgM and mIgD BCR with high affinity for hen egg lysozyme (HEL). When these mice were crossed to ML5 mice expressing soluble HEL, B cells developed and occupied peripheral lymphoid organs, but the animals were unresponsive to immunization (9, 18). Transfer of naïve MD4 B cells or MD4xML5 B cells to B6 recipients with CD4 T cells that recognize sheep red blood cells (SRBCs), followed by immunization with HEL-SRBCs, led to a response by both populations, though the response of the latter cells was greatly reduced. MD4xML5 cells did not respond when transferred to ML5 recipients. The former must reflect gradual dissociation of autoantigen from BCR following transfer to the autoantigen free environment, with attendant loss of unresponsiveness, or “anergy” (19). The inability of anergic B cells to mount an equivalent immune response following transfer could be due to an inability of cells to cooperate with cognate T cells, due either to failure to process and present antigen or to respond to T cell derived signals. To determine whether the defect in these anergic cells lay in the ability to internalize, process, and present antigen to T cells, they were modified to constitutively express MHC class II with peptide, bypassing the need to process and present antigen, and allowing interaction with cognate T helper cells (7). If the defect lay only in antigen processing and presentation, adoptive transfer of these B cells into B6 or ML5 recipient mice, followed by immunization, should have led to an immune response. However, these MD4xML5 anergic B cells failed to respond by producing anti-HEL antibodies (7). Interestingly, naïve MD4 B cells adoptively transferred into ML5 recipients responded to antigen by proliferation and differentiation. This evidence suggests that the immune response defect in anergic B cells must reflect more than an inability to process and present antigen. To determine the ability of anergic B cells to respond to T cell help, naïve MD4 B cells and anergic MD4xML5 B cells were stimulated *in vitro* with IL-4 and anti-CD40 and responses assessed. Both naive MD4 B cells and anergic MD4xML5 B cells upregulated MHC class II and costimulatory molecules, i.e., CD86, in response to these stimuli that mimic T cell help (7, 8). These data demonstrated the reversibility of anergy, as well as suggest there is not an inherent defect in the ability of an anergic B cell to respond to T cell help. They left open the possibility that the defect could lie in an inability of the anergic cell to upregulate T cell costimulatory ligands such as CD86 in response to antigen.

Because the previous experiments indicated that the inability of anergic B cells to respond to antigen is not limited to an antigen processing and presentation defect, it seemed likely that there was defect(s) in antigen receptor signaling. To determine the ability of anergic B cells to respond to BCR ligation, *in vitro* responses of naïve MD4 B cells and anergic MD4xML5 B cells were compared. Unlike naïve cells, MD4xML5 failed to proliferate, increase RNA synthesis indicative of entry into cell cycle, or upregulate CD86 (7). These data suggest that there is an inherent defect in the ability of an anergic B cell to signal through their antigen receptors. Confirming this, anergic B cells failed to mobilize calcium in response to BCR stimulation. Antigen stimulation of anergic B cells did not lead to a significant increase in protein phosphorylation (7). Tolerant B cells show a decrease in cell surface IgM antigen receptors, possibly explaining the decrease in signaling. However, anergic B cells transferred into B6 recipients and “parked” for 36 h led to normalization of receptor levels and equivalent fluorescently labeled antigen binding, but the cells remained unresponsive to antigen based on calcium mobilization (7). It is important to note that while anergic B cells downregulate mIgM, they do not downregulate mIgD, which constitutes 90% of the antigen-binding capacity of most splenic B cells (20). This alone would argue that hyporesponsiveness of anergic B cells is not attributable to reduced antigen-binding capacity.

Protein tyrosine phosphorylation is the earliest quantified event in BCR signaling. Loss of this event in anergic cells suggests that unresponsiveness may reflect a defect in initial transduction of signals across the plasma membrane (7, 21). Consistent with this possibility, it has been reported that antigen stimulation can lead to rapid destabilization of the interaction of mIgM with the CD79a/b (Igα/β) heterodimer (22). Reductionist studies using B cell lines ectopically expressing association-competent versus incompetent BCR demonstrated that incompetent BCRs can compromise competent receptor signaling within the same aggregate/complex. In fact, receptor complexes containing as few as 13% incompetent CD79-associated mIg showed defects in signaling (22). Thus, mechanisms that act to limit BCR signaling in anergic cells may somehow target the structural integrity of the antigen receptor itself.

The discussion above describes extant knowledge of biological and BCR signaling defects associated with B cell anergy in the MD4 anti-HEL model. The findings described were confirmed in another model, the Ars/A1 model, in which B cells are reactive with chromatin (13). Below, we drill more deeply into proximal BCR signaling pathways and negative regulatory mechanisms that limit the antigen responsiveness of anergic cells.

## Antigen Receptor Signaling in Naïve and Anergic B Cells

**Figure 1:**
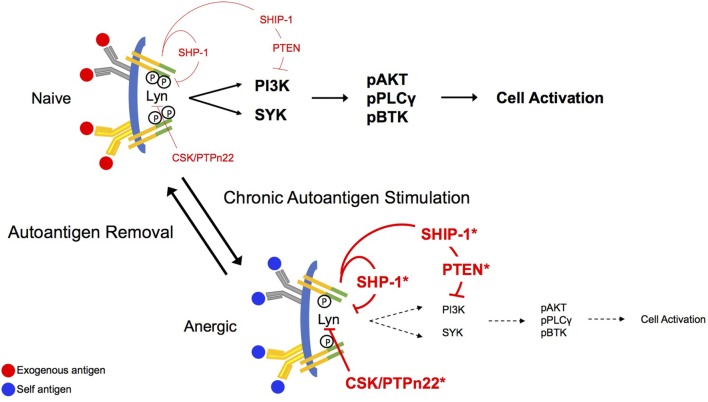
Antigen receptor signaling in naïve and anergic B cells. B cell antigen receptor (BCR) aggregation in naïve cells leads proximally to phosphorylation of CD79a and CD79b immunoreceptor tyrosine-based activation motif (ITAM) tyrosines. This phosphorylation allows recruitment of the Src-family kinase Lyn to the receptor where it binds through its SH2 domain and its activity is derepressed. Lyn mediates activation of the phosphoinositide 3-kinase (PI3-kinase) pathway *via* phosphorylation of CD19, and the PI(3,4,5)P3 product of this pathway sequesters BLNK, PLCγ, and BTK for function in signaling. Phosphorylation of both ITAM tyrosines leads to binding of SYK *via* its dual SH2 domains and to subsequent activation. Under conditions of chronic antigen stimulation (anergy), the ITAMs on CD79a are preferentially monophosphorylated, recruiting Lyn to the complex. Lyn, in its negative regulatory role, recruits the function of the inositol phosphatases, SHIP-1 and PTEN, as well as the tyrosine phosphatase, SHP-1. These regulatory phosphatases will then exert their inhibitory function on downstream effector molecules, abrogating stimulatory BCR signaling. Bias toward stimulation of inhibitory circuitry is reversed upon removal of the antigen receptor stimulus.

In naïve B cells, BCR stimulation leads most proximally to the tyrosine phosphorylation of two conserved tyrosine residues embedded in immunoreceptor tyrosine-based activation motifs (ITAMs) found in CD79a and CD79b, the heterodimeric signaling component of the BCR, as indicated in Figure 1 (23–26). This phosphorylation appears to be governed by the balanced activity of phosphotyrosine phosphatases and SRC family kinases for which ITAMs are substrates (27–29). Phosphorylated ITAMs stimulate Lyn activation, presumably through association with the kinase SH2 binding and derepression of its enzymatic activity (30). ITAM bi-phosphorylation enables receptor binding of the Syk tyrosine kinase *via* its dual SH2 domains leading to its phosphorylation and activation (28, 31). BCR stimulation leads to concurrent Lyn-mediated tyrosine phosphorylation of CD19, a BCR accessory/co-receptor, enabling its association with Lyn and phosphoinositide 3-kinase (PI3-kinase) (32). CD19, functioning in conjunction with the adaptor BCAP, mediates activation of PI3-kinase and generation of PI(3,4,5)P3 (33). The head group of this inner leaflet phospholipid second messenger binds the plecktrin homology (PH) domains of a number of critical downstream effectors, including PLCγ, AKT, PDK1, and BTK, localizing them to the receptor where they can be activated by phosphorylation (31). Multiple parallel pathways diverge from this activated receptosome, leading ultimately to cell activation.

Anergic B cells are chronically stimulated by autoantigen *in vivo*, but at least in MD4xML5 and Ars/A1 models, cell surface BCRs are not saturated. Indeed, on immediately *ex vivo* anergic cells most receptors are unoccupied (13). Additionally, chronic *in vivo* autoantigen stimulation results in monophosphorylation of receptor ITAMs (16). Findings from cell-free experiments in which the ability of specific kinases to phosphorylate CD79 ITAMs was assessed demonstrate that Lyn and Fyn, but not Syk efficiently phosphorylate these substrates. However, this phosphorylation occurs primarily on the more N-terminal ITAM tyrosines, and ITAM bi-phosphorylation is a minor event (~20%) (28, 30). This may indicate that the degree of ITAM tyrosine phosphorylation is highly regulated by factors in addition to degree of aggregation. In terms of downstream consequences, it should be noted that because activation of Syk requires binding of both SH2 domains to phosphorylated ITAM tyrosines, monophosphorylation is associated with Lyn, but not Syk activation (30). Consistent with this, induced phosphorylation of the Lyn substrate CD19 appears normal (Getahun and Cambier, unpublished) in anergic cells, but pathways downstream from Syk are silent. Phosphorylation of CD19 suggests that PI3-kinase is active in these cells. Anergic B cells exhibit increased basal and BCR-mediated tyrosine phosphorylation of the PI(3,4,5)P3 5-phosphatase SHIP-1 and its adaptor Dok-1 (34), previously shown to be associated with their activation (11, 35). They are also characterized by an increase in expression of PTEN, a PI(3,4,5)P3 3-phosphatase, that is subject to regulation by a number of microRNAs (36, 37).

## Regulation of BCR Signaling in Naïve and Anergic B Cells

### Lyn

Interestingly, Lyn, the primary BCR-associated Src-family kinase, plays both positive and negative functional roles in antigen receptor signaling (38–40). Allelic differences in the LYN gene leading to reduced expression of the kinase confer increased risk of developing SLE, and patients with lupus have decreased Lyn expression in B cells (41, 42). In mice, Lyn deficiency increases negative selection in the bone marrow with fewer Lyn^−/−^ B cells being found in the periphery of Lyn^−/−^ MD4 mice (43). Peripheral Lyn^−/−^ B cells fail to fully mature (43, 44). Lyn^−/−^ B cells exhibit delayed but exaggerated and more sustained calcium response to antigen (39, 45), further suggesting both positive and negative roles in BCR signaling. In the absence of Lyn, other Src-family kinases expressed in B cells (Blk and Fyn) act to propagate BCR signaling, but down modulation of BCR signaling is abrogated, suggesting loss of anergy. The balance of Lyn and Fyn is further explained *in vivo* with Lyn deficiency exacerbating nephritis and arthritis, while loss of Fyn is protective from auto/inflammatory disease (46). Moreover, patients with SLE present in the clinic with a Fyn-activating signature, further suggesting a negative role for Lyn in BCR signaling.

Lyn^−/−^ mice develop an SLE-like disease as indicated by autoantibody production and glomerulonephritis (47). B cells from these mice undergo enhanced proliferation in response to BCR crosslinking. Macrophages and dendritic cells also play a role in development of disease in Lyn^−/−^ mice. However, B cell-specific conditional deletion of Lyn, achieved by crossing the Lyn^fl/fl^ mouse to a mouse carrying Cre expressed under the CD79a promoter, leads to autoantibody production, IgG immune complex deposition ultimately resulting in glomerulonephritis (44).

Lyn may exert its negative regulatory function through phosphorylation of immunoreceptor tyrosine-based inhibitory motifs (ITIMs) on CD22, leading to recruitment of SHP-1 (48), a tyrosine phosphatase that dephosphorylates activating signaling molecules, such as CD79a/b ITAMs, Syk, and BLNK (49, 50). Lyn also drives inhibitory signaling by promoting the inhibition of the PI3-kinase pathway, by phosphorylating the inositol phosphatase, SHIP-1 and its Doc family adaptors, Doc-1 and Doc-3. SHIP-1 hydrolyzes PI(3,4,5)P3 yielding PI(3,4)P2, preventing recruitment, and activation of PH domain-containing effectors and consequent propagation of BCR signals. Alleles of genes that encode components of this regulatory axis, including SHP-1 (38, 51), Csk (52), PTPn22 (53–56), and Lyn (41), have been shown to confer risk of autoimmunity (57). Reduced PTEN and SHIP-1 levels presumably caused by increased in expression of microRNAs that regulate them are also seen in autoimmunity. Demonstrating their critical roles in maintenance of anergy, we have shown that acute deletion of these proteins from anergic B cells *in vivo* results in rapid cell proliferation and differentiation, and production of autoantibodies.

### SHP-1

An allele of the regulatory SH2-containing tyrosine phosphatase SHP-1 has been associated with increased risk of developing SLE (38). SHP-1 and SHP-2 mediate the function of certain inhibitory ITIM containing receptors, such as CD22, PD1, and FcγRIIB, although there is evidence that SHP-1 is dispensable for the latter (58, 59). SHP-1 was first described as being crucial for FCγRIIB-mediated negative regulation of anti-BCR induced proliferation in motheaten mice (me) (60). The ultimate resolution of this inconsistency came from studies of Lasourne and colleagues who showed that the degree of packing of phosphorylated FcγRIIB ITIMs determined the relative involvement of SHIP-1 and SHP-1 in downstream inhibitory signaling. Higher level aggregation, as probably occurred in the D’Ambrosio studies, would be expected to evoke SHP-1 function.

Viable motheaten mice (me^v^/me^v^) have a mutation that interferes with a splice site in the gene that encodes SHP-1, *Ptpn6*, reducing the enzyme activity to 10–20% of wild type. These animals exhibit severe B cell immunodeficiency and autoantibody production. In me^v^ crossed to the MD4, SHP-1 low B cells undergo increased intracellular calcium flux responses to antigen. SHP-1 deficiency also leads to increased serum levels of IgM, IgG_1_, and IgG_3_ (61). Furthermore, B cell-specific loss of SHP-1 leads to an accumulation of B-1a cells and systemic autoimmunity (62, 63). Acute B cell targeted deletion of SHP-1 from anergic B cells *in vivo* leads to cell activation, proliferation, differentiation to plasmablasts and autoantibody production (62). Genetic complementation studies indicate that SHIP-1 and SHP-1 act in distinct regulatory pathways both of which must be functional to maintain anergy.

Studies indicate that in a subset of SLE patients, B cells express reduced SHP-1 protein, suggesting these patients have a decreased ability to maintain B cell anergy (51). A decrease in SHP-1 (both protein and mRNA) in PBMCs isolated from multiple sclerosis patients is associated with an increase in inflammatory gene expression (64). This is particularly interesting given both me^v^ mice and the inducible mouse model of MS, experimental autoimmune encephalomyelitis, have more severe disease when SHP-1 deficiency is observed (65, 66).

### SHIP-1 and PTEN: Cooperative Enforcement of Anergy

Phosphoinositide 3-kinases function in the promotion of numerous biological functions by the generation of lipid second messengers. Mice that lack the dominant PI3-kinase isoform found in B cells, p110δ, show a reduction in calcium mobilization, a decrease in serum immunoglobulin levels, as well as a decrease in germinal center formation in the spleen (67, 68). Tight regulation of this pathway by the inositol phosphatases, SHIP-1 and PTEN, is critical for maintaining tolerance to self-antigens.

Studies utilizing adoptive transfer of anergic B cells, followed by targeted deletion of SHIP-1, have shown that this inositol phosphatase is crucial for maintenance of anergic B cell unresponsiveness to self-antigen. Upon deletion of SHIP-1, B cells that were once anergic become activated, upregulating costimulatory molecules, and proliferate and differentiate into antibody secreting cells (62). Furthermore, B cell targeted deletion of SHIP-1 results in systemic autoimmunity (16, 69). In addition to its enzymatic activity, SHIP-1 functions as an adaptor protein, binding effectors such as rasGAP (34). To determine if SHIP-1’s role in maintaining anergy is a function of its regulation of the PI3-kinase pathway as opposed to its adaptor functions, Getahun et al. used genetic models to conditionally delete PTEN or express a constitutive active PI3-kinase p110α in anergic B cells *in vivo* (62). Loss of PTEN expression or enforced over-production of PI(3,4,5)P3 led to a breach of anergy. Interestingly, while haploinsufficiency of either SHIP-1 or PTEN alone does not lead to loss of anergy, haploinsufficiency of both inositol phosphatases does, consistent with the fact that these phosphatases act in the same regulatory pathway.

SHIP-1 levels are decreased in Fas^MRL/lpr^ mice due to an increase in the microRNA that regulates its expression, microRNA 155 (36). microRNA 155 is also elevated in SLE patients and has been correlated with disease activity (70). Additionally, descriptions of decreased PTEN expression, caused by an increase in expression of microRNA 7, in B cells from patients with SLE have been correlated with disease severity (37). These data confirm that induced inhibition of the PI3-kinase pathway is critical for maintaining tolerance and preventing anergic B cells from participating in an autoimmune response. They further implicate microRNA levels as indirect regulators of anergy.

### Csk/PTPn22

PTPn22 variants are among the risk alleles most strongly linked to human autoimmunity, but the molecular mechanism of PTPn22 action and consequence of risk allele defect(s) remains unclear. The PTPn22 R620W allele is found at high frequency in patients with type 1 diabetes, RA, SLE, Grave’s thyroiditis, and myasthenia gravis, but does not predispose to other diseases, such as multiple sclerosis, Crohn’s disease, or psoriasis vulgaris. While there are additional PTPn22 risk alleles that are associated with increased risk of autoimmunity (71), we will focus on the more understood R620W allele.

PTPn22 is a nonreceptor tyrosine kinase that binds Csk, a known suppressor of antigen receptor signaling. PTPn22 has also been shown to negatively regulate signaling by dephosphorylating Src family kinases (72, 73). PTPn22^−/−^ mice develop increased serum immunoglobulin levels and germinal centers although overt autoimmunity is absent. The R620 is located in a position critical for binding of PTPn22 to Csk, suggesting that R620W would decrease the interaction of the two molecules, possibly resulting in increased antigen receptor signaling. However, Liston et al. demonstrated R620W causes reduced TCR signaling, leading to reduced thymic selection and subsequent deletion, allowing for more autoreactive T cells to exit into the periphery to later become activated and participate in an autoimmune response (74). Furthermore, R620W human B cells have decreased BCR signaling and BCR-mediated responses, but have an increased autoreactive B cell compartment, coupled with less effective central and peripheral tolerance. Patients who carry the mutated form of PTPn22 have an increased autoreactive B cell compartment, coupled with less effective central and peripheral tolerance. This is also seen in mice that express the human equivalent mutation, R619W, in B cells only. These mice develop autoimmunity (75).

## Concluding Remarks

This review summarizes a body of work that has defined B cell anergy and the molecular mechanisms that maintain antigen unresponsiveness of anergic B cells. It further describes the effects of allelic variations of regulatory signaling molecules that confer increased risk of autoimmunity. Role for these risk alleles in failed silencing of autoreactive B cells *per se*, underscore the potential of targeting B cells for therapeutic intervention in autoimmunity [reviewed in Ref. (76)]. In the era of precision medicine, therapy will be based on genetics in addition to symptomology. As with all therapies for autoimmunities, there is a balance to be struck between controlling the autoimmune response while still leaving patients competent to mount protective immune responses to pathogens.

## Author Contributions

SEF and JCC both participated in the writing and preparation of the manuscript.

## Conflict of Interest Statement

The authors declare that the research was conducted in the absence of any commercial or financial relationships that could be construed as a potential conflict of interest.
